# The Influence of Weekly Sprint Volume and Maximal Velocity Exposures on Eccentric Hamstring Strength in Professional Football Players

**DOI:** 10.3390/sports10080125

**Published:** 2022-08-19

**Authors:** Sunnan Shah, Kieran Collins, Lewis J. Macgregor

**Affiliations:** 1 Faculty of Health Sciences and Sport, University of Stirling, Stirling FK9 4LA, UK; 2 Gaelic Sports Research Centre, Institute of Technology Tallaght, D24 FKT9 Dublin, Ireland

**Keywords:** injury risk, sprint distance, sprint efforts, strength and conditioning, Nordic Hamstring Exercise, NordBord, performance monitoring, GPS

## Abstract

Background: Hamstring strains are the most common moderate-major severity injuries in football. The majority of hamstring injuries occur during sprinting, with low eccentric hamstring strength being associated with an elevated risk. Objective: To examine the relationship between sprinting and eccentric hamstring strength by monitoring total weekly sprint distance and weekly efforts > 90% and >95% of maximum velocity. Methods: Fifty-eight professional male footballers were observed over one-and-a-half seasons. Players’ running was monitored during training and matches using GPS, and eccentric hamstring strength was measured weekly. Results: Weekly sprint distance (ρ = −0.13, *p* < 0.01) and weekly efforts >90% of maximum velocity (ρ = −0.08, *p* = 0.01) both displayed significant inverse relationships with the percentage change in eccentric hamstring strength; weekly efforts >95% of maximum velocity showed no relationship with hamstring strength (ρ = −0.02, *p* = 0.45). Only weekly efforts >90% of maximum velocity significantly influenced the mean percentage change in eccentric hamstring force, F_(3,58)_ = 3.71, *p* = 0.01, with significant differences occurring when comparing 7–8 sprint efforts with 0–2 efforts (0.11%, *p* = 0.03) and 5–6 efforts (0.12%, *p* = 0.03). Conclusions: Eccentric hamstring strength levels significantly decrease when 7–8 weekly sprint efforts are completed at >90% of maximum velocity. Monitoring weekly sprint loading at velocities > 90% of maximum velocity may be valuable to help to reduce the risk of hamstring injuries in professional football.

## 1. Introduction

Within football, musculoskeletal injuries account for 97% of all injuries sustained, with 87% of those injuries occurring in the lower extremities [1]. Hamstring strains are the most common of these injuries resulting in moderate and major injury severity, defined as 8–28 days and >28 consecutive days injured in a season, respectively [1,2,3]. With a typical football season consisting of 40–50 competitive matches, losing more than 28 consecutive days can be significant for teams as a player may miss 4–8 matches. The players themselves could suffer a loss of earnings, and potential long-term health issues, including disability and forced early retirement. Additionally, the risk of a hamstring re-injury rises by 13.9–63.3% within the first two years of returning to play, with the associated time loss also increasing depending on the severity of the initial injury [4,5]. The implications and prevalence of hamstring injuries in football necessitate greater understanding of the mechanisms involved, to better inform preventative measures.

Hamstring strains are stretch-induced injuries, meaning that they occur when the muscle is lengthened passively, or activated during a stretch—referred to as eccentric contraction [6]. Muscle strains typically occur when external forces applied to a muscle exceed the force produced by the muscle itself. Eccentric contractions are associated with high forces coupled with fewer active motor units [7]. Due to high eccentric forces involved, sprinting is the primary mechanism of hamstring strains, accounting for 57% of all hamstring injuries [8]. Sprinting plays a key role in professional football, where it has been found to be the most frequent action involved in goal-scoring situations [9]. Furthermore, the amount of high-velocity running and sprinting, essential in elite level football, increased by 24–36% between seasons 2006/07 and 2012/13 [10,11]. This increase in high-velocity running and sprinting over time may partly explain the observed rise in hamstring injuries, with prevalence increasing by 4% per year from 2001 to 2014 [12]. As well as highlighting the trends between sprinting and its associated injuries, these findings show the importance of the hamstring muscles during high-velocity running. The hamstrings play a crucial role in producing horizontal force and in energy absorption, and are therefore a key muscle group when running at high velocities [13,14]. During sprinting, the hamstrings are highly activated in the early stance phase where initial contact occurs and, in particular, during the late swing phase [15,16]. During the late swing phase, activation of the hamstrings is found to be two to three times greater than the earlier phases of sprinting [15]; the hamstrings undergo eccentric contraction during this phase, which involves the lengthening of the muscles during contraction and absorbing the mechanical work being completed [15,17]. Although sprinting is the primary mechanism of hamstring strains, there are many contributing factors that must be considered to help to reduce the risk of injury; while factors such as age, race or previous injury cannot be modified, there remain a number of modifiable factors [15].

The modifiable factors involved in hamstring strains include a lack of muscle flexibility, strength imbalance between limbs, quadriceps-to-hamstring strength ratio, insufficient warm-up, and fatigue [15,18]. The numerous factors influencing hamstring injury mean that the term “injury risk” can be multi-factorial and therefore extremely difficult to quantify. For this reason, previous studies have isolated individual factors in order to understand their effect on hamstring injury [18,19]. The majority of the factors mentioned have an overall effect on ‘hamstring strength’, where the reduced strength of the hamstring muscles is found to correlate with injury occurrence during the late swing phase as the muscles are not strong enough to counteract the contractile forces produced by the quadriceps [19]. Additionally, professional football players with eccentric hamstring peak torque less than 2.44 times their bodyweight and a quadriceps-to-hamstring ratio lower than 50.5% show increased injury risk by 5.6-fold and 3-fold, respectively [18]. Elsewhere, eccentric hamstring force below 337 N has been found to increase the risk of hamstring injury by 4.4-fold [20]. The common conclusion is that improving hamstring strength is paramount to reducing the risk of hamstring injury. Therefore, it is important to consider methods to increase hamstring strength in a professional training environment.

Exercises that focus on eccentric contraction have been shown to improve hamstring strength more effectively than concentric exercises [21]. Eccentric exercises such as the Romanian Deadlift, Good Morning and Glute Ham Raise have been shown to illicit high activation of the hamstrings [22], while the Nordic Hamstring Exercise (NHE) has been found to result in architectural adaptations and is effective in improving eccentric hamstring strength [21,23,24,25,26,27]. In addition to being effective in improving eccentric hamstring strength, the NHE can be easily applied in the practical setting of a sports team due to its simplicity. Advancements in technology have also helped practitioners prescribe the NHE using specialised apparatus, such as the Nordbord by Vald Performance and the Hamstring Solo Elite by ND Sports Performance, which can be used to give instant feedback on the force produced by each limb’s hamstring muscles. The NHE involves the knee flexors working eccentrically to control knee extension. This action mimics the movement that occurs during the late swing phase of sprinting and has therefore been shown to be a valid measure of knee flexor strength [28,29]. Single eccentric strength assessments based on NHE performance—such as might be conducted during the pre-season—are insufficient to predict future hamstring injury occurrence [30,31], therefore, continuous monitoring is recommended. One of the benefits of the NHE is that the hamstrings contract over a large range of motion where the knee is initially flexed at 90° and extends towards 0° (full extension) at the end of the movement. Peak torque, which combines the force and length of the lever arm, during the NHE has been found to occur between 18–28° of knee flexion, where the hamstrings are lengthened while contracting [32]. These angles are similar to those found during the late swing phase of sprinting, further linking the NHE to high-velocity running [33].

While the development of hamstring eccentric strength is important to reduce the risk of injury, it is also vital to stimulate the muscles by providing frequent exposures to high velocities in order to provide a training effect [34]. This may appear surprising since sprinting is the primary mechanism for hamstring injuries; however, with the nature of football relying on high-velocity running, it is important that players receive adequate stimulus when training to meet the demands of a competitive match. To prepare athletes tactically and physically, consistent exposure to match situations is key; hamstring injury rate is nine times higher during a competition, when compared with training [12]. Infrequent or over-exposure to a stimulus is considered to be a ‘spike’ in an athlete’s workload, which is found to be a risk factor for injury [35,36]. These spikes in training have been highlighted by the work on the acute:chronic workload ratio (ACWR), which looks into an athlete’s short-term training workload compared to their long-term workload to identify any large increases in the work completed [35]. It is important that the players are overloaded to gain a training response; however, a gradual overload is required to avoid any spikes in workload which may result in an increased risk of injury [37].

During football training, a key emphasis is placed on small-sided games due to their ability to mimic the situations and intensities found in a match [38]. However, due to restricted space, these games tend to involve relatively few high-velocity running exposures. Therefore, supplementing training with high-velocity linear running may offer a solution to help to manage athlete workloads and avoid spikes on match-days. It has been found in Gaelic and Australian Rules Football that frequent exposure to high-velocity running reduces the risk of hamstring injuries [34,36]. Exposure to velocities greater than 95% of a players’ maximum sprint speed (max_>95%_) even once per week during training can lower the risk of injury, with 6–10 exposures found to be optimal for minimising injury risk [34]. These values are relative to the physical demands of Gaelic football, where during a match, players typically complete 44 sprint actions corresponding to a higher sprint distance than football (soccer) (445 ± 169 m versus 285 ± 115 m, respectively) [39,40,41,42]. Due to the lower sprinting demands in football during a match, maximal efforts at max_>95%_ would need to be supplemented during training, but this is difficult to achieve within the practical setting of football due to lower motivation levels and freshness in training compared with match conditions [43]. Therefore, being exposed to velocities above 90% of an individual’s maximum sprint speed (max_>90%_) would be more achievable in football training. Malone et al. [34] did not find efforts <95% of maximum sprint speed to be as beneficial in reducing injury risk; however, Colby et al. [36] found that 5–8 efforts at 85% of a player’s maximum sprint speed reduced injury risk in Australian Rules Football. Therefore, for the purpose of this study max_>90%_ was used. Since the original study by Malone et al., which looked at Gaelic football, further research has followed on football (soccer) [44]. Similar to the previous study, the risk of injury was found to increase with insufficient or excessive high-velocity stimulation. However, in that study, distances were investigated rather than individual exposures, where 701–750 m of high-speed running and 201–350 m of sprint distance per week were shown to reduce injury (odds ratio = 0.12 and 0.54, respectively) [44].

Therefore, our overall objective was to examine eccentric hamstring strength, one of the factors contributing towards hamstring injury risk, with respect to the effects of maximal velocity running. The aims of the study were to investigate the relationship between hamstring force output during NHE performance and (1) total weekly sprint distance (m) and (2) the number of exposures above 90% of maximum velocity (max_>90%_). We aimed to determine the optimal total weekly sprint distance and number of exposures at max_>90%_ required to illicit peak eccentric force output. In addition, we aimed to determine whether the total sprint meterage or the number of individual exposures at max_>90%_ had the largest effect on force output during the NHE. Findings from this study could be applied within a professional football environment to help prevent injuries associated with hamstring strength.

## 2. Materials and Methods

### 2.1. Participants

Fifty-eight male players from a professional football team took part in the study (Table 1). Within this sample, 20 were measured over the course of one-and-a-half football seasons (July 2018–January 2020), 14 were measured over the course of one full football season (July 2018–May 2019) and 24 players were measured over the course of half a football season (July–January or January–May between 2018–2020). Any players that could only be measured for less than half of a season, such as short-term loans and players with long-term injuries, were omitted from the study. Goalkeepers were also omitted from the study due to the different nature of their activity. Players were categorised as ‘defenders’, ‘midfielders’ or ‘attackers’ based on the position they played most frequently during the study period. Players who played as centre backs, full backs or wing backs were regarded as defenders. Players who played as central midfielders, whether defensive or attacking, or right and left midfielders were regarded as midfielders. Players who played as strikers or wingers were regarded as attackers. The reason for grouping the players into three general positions was for simplicity and to account for any ambiguity based on slight differences in formations and roles. The participants were all full-time professional athletes, training at least three days per week, from the elite and development squads. All players played competitive fixtures at their respective age groups, including the Scottish Premier League, Reserve League and the Under 18’s League.

Before the start of the study, participants received an information sheet detailing the purpose, potential risks and benefits of the study before providing written informed consent. This study was approved by the NHS, Invasive or Clinical Research Committee (NICR) at the University of Stirling (18/19-004).

### 2.2. Sprint Monitoring

Players’ movements were monitored during training sessions and matches. Training session data was captured daily, from the start of the warm-up to the end of the session. Match data was captured from kick-off to the final whistle (or the total duration of the players’ involvement, if they were substituted on or off the pitch during the match). Training and match data were monitored using global positioning system (GPS) devices designed to measure external load (Catapult Optimeye X4, 2.4 GHz RF Device, Catapult Sports, Melbourne, Australia). These devices had a sampling rate of 10 Hz and the velocity dwell time (minimum duration of effort) was set to 0.6 s, for consistency with previous data held at the football club [45]. On average, there were eight satellites connected to the devices during training and the matches, suggesting that the quality of data was sufficient [46]. GPS units were worn in Catapult vests, specifically designed to hold the unit between the shoulder blades and limit movement. It was important that the device was placed in the vest correctly, ensuring the unit was not inserted at an angle and the power button was facing the outside to improve reliability of the satellite signal and the movements recorded. These data were initially downloaded and analysed using the manufacturer’s software (Catapult Sport’s Openfield Console and Openfield Cloud) before being exported to Microsoft Excel Version 16.16.27 (Microsoft Corporation, Redmond, WA, USA) for further analysis. For this study, we recorded each player’s sprint distance (m) and maximum velocity (m/s) for each session, and the number of sprint efforts > 90% and >95% of maximum velocity. For distance measurements, 10 Hz GPS devices have previously demonstrated standard errors of measurement of 5.1–10.9% and coefficients of variation of 0.7–1.3% for short distance sprints [47], and coefficients of variation of 1.9–4.7% for longer distance sprints [48]. For velocity assessment, specifically during team sport simulation, 10 Hz devices have demonstrated technical error in measurement and intraclass correlation coefficient of 1.6% and 0.97, respectively [49]. Other than sprint distance, which was a pre-set parameter on the software, these parameters were created manually on the Openfield Cloud. These values were then combined across each day to present weekly totals. All weekly totals, which included all training sessions and matches, were then analysed with the corresponding NHE scores for that week. For sessions where data were not obtainable as a result of the players not wearing or turning on the GPS unit, the unit cutting out due to battery issues, or the data being unreliable due to an intermittent satellite signal, estimations were used. For training data, these estimations were taken, as an average, from the other players of a similar position who completed the same drills within the session. For match data, these estimations were taken, as an average, from the individual’s previous five matches [50].

### 2.3. Nordic Hamstring Exercise

The players were required to perform one set of three repetitions of the NHE per week for the purposes of strength development and monitoring neuromuscular status. These were primarily performed two days after a match (MD+2) as part of the players’ routine strength and conditioning programme. Strength and conditioning sessions were completed in the morning before any other training, with the NHE being the first exercise in the programme to avoid being in a fatigued state when performing the NHE so that valid scores could be obtained. As it was the first exercise, the players were required to perform one set of three repetitions of the NHE at 50% effort as a warm-up, followed by one set of three repetitions at maximal effort. Each repetition was performed on a hamstring testing device specifically designed for performing the NHE (NordBord Hamstring Testing System, 50 Hz, Vald Performance, Brisbane, QLD, Australia), which was used to measure bilateral force output (N) and between limb strength imbalances during the lowering eccentric phase of the NHE. The exercise was recorded on a Windows laptop or iOS device using the manufacturer’s live software (ScoreBord) and then uploaded to the manufacturer’s online platform (Dashboard), where it was then exported to Microsoft Excel Version 16.16.27 (Microsoft Corporation, Redmond, WA, USA) for further analysis. Specifically, for the purpose of the study, weekly absolute peak bilateral force (N) scores for each player were analysed alongside the sprint data for that corresponding week. Absolute peak bilateral force output (N), which was the highest force, from the three repetitions, exerted by the hamstrings when performing the NHE, was measured by the load cells attached to the hooks of the NordBord. As the hooks were unilateral, each limb’s force output could also be calculated by the ScoreBord software to provide limb asymmetry (%). Before commencing the study, we assessed reliability and validity of the NordBord; we found intra-day and inter-day CV of 10% and 11%, respectively, and ICC of 0.80 [95% CI: 0.21, 0.96] and 0.95 [95% CI: 0.74, 0.99], respectively. When analysing the validity of the NordBord against the “gold standard” method of testing muscle strength—the isokinetic dynamometer—correlation coefficients of *r* = −0.074 (*p* = 0.053), *r* = 0.147 (*p* = 0.002) and *r* = −0.047 (*p* = 0.007) were obtained during isometric testing on the left, right and both limbs, respectively, while *r* = 0.530 (*p* = 0.017), *r* = 0.444 (*p* = 0.247) and *r* = 0.528 (*p* = 0.044) were obtained for eccentric strength.

**Figure 1:**
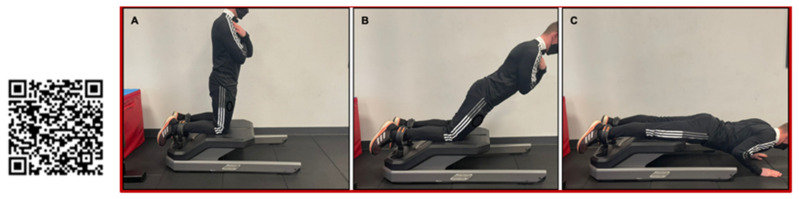
Performing the Nordic Hamstring Exercise on NordBord. (**A**) Starting in upright position, (**B**) contracting knee flexors during movement to control descent, (**C**) finishing by placing hands on the ground after breaking point; all while ankles are secured. Scan QR Code for video of NHE.

The protocol of the NHE involved the player placing their heels under the hooks with their knees placed on the NordBord. Their knee position was recorded in the software for consistency during every repetition each time the exercise was performed. For the starting position, the player’s knees began at 90°. Once in position, the player was then required to lower their torso in a controlled manner over a minimum of three seconds, until they could no longer hold the movement. During this movement, the players were encouraged to keep their shoulders, hips and knees in line through verbal cues given by the practitioner so that neutral hip alignment was maintained. They would then catch themselves at the end of the movement by placing their hands on the floor, walk their hands back in and, when ready, repeat to complete three repetitions (Figure 1). The players were allowed a maximum of three minutes to complete all three repetitions, with most players requiring less than one minute.

The NHE can require a thorough familiarisation period due to the complexity of the exercise. Therefore, the players’ data were only recorded once they had completed a minimum of three weeks of the exercise protocol; however, many of the players had experience with using the NordBord prior to the study, so their data were recorded from the beginning of the sampling period. When it was impossible to test a player’s NHE due to scheduling issues, managing the player’s training load, or for any other reason, that corresponding week was removed from the analysis.

### 2.4. Statistical Analysis

All data were initially exported to Microsoft Excel Version 16.16.27 (Microsoft Corporation, Redmond, WA, USA) for the first stage of analysis. At this stage, the data were sorted and filtered based on the criteria described above. Relative change in peak bilateral eccentric hamstring strength, measured during NHE, was calculated for each player to account for inter-individual strength differences. For each player, peak bilateral eccentric strength was calculated as the percentage change from the baseline. Baseline peak bilateral strength was taken from the point when players could be considered suitably familiar with NHE—familiarity was established when the mean of three consecutive NHE tests elicited a coefficient of variation <10%, based on our previous reliability findings. Further analysis was then completed on SPSS Statistics Version 26 (IBM Corporation, New York, NY, USA). A correlation coefficient analysis using Spearman’s rho was used to measure the degree of association between total weekly sprint distance (m), efforts > 90% and >95% of each player’s maximum velocity and their effect on eccentric hamstring strength; correlations with *p* < 0.05 were deemed significant. A correlation (ρ) less than 0.30 was considered small; 0.31 to 0.49 moderate; 0.5 to 0.69 large; 0.70 to 0.89 very large; and 0.90 and higher near perfect [51]. These thresholds also applied to negative values, indicating an inverse correlation. A one-way ANOVA with Tukey’s post hoc test was used to determine any significant differences between the total weekly sprint distance, efforts > 90% and efforts > 95% on the mean percentage change in eccentric hamstring force.

## 3. Results

### 3.1. Participant Data

The mean maximum velocity (*n* = 58 players) was 9.27 ± 0.27 m/s. Over the course of the study, players completed 209,139 m of sprint distance, 947 efforts and 16 efforts > 90% and >95% of maximum velocity, respectively. Per week players covered 212.1 ± 188.6 m, including 0.96 ± 1.39 efforts and 0.02 ± 0.14 efforts > 90% and >95% of maximum velocity, respectively (Table 2). Mean hamstring force output was 427.47 ± 57.98 N (SEM = 1.85) with mean strength imbalance of 8.20 ± 6.65% (SEM = 0.21) between limbs (Table 2).

### 3.2. Relationship between Sprinting and Hamstring Strength

A significant inverse relationship between total weekly sprint distance and percentage change in eccentric hamstring strength was found, with a very small correlation shown (ρ = −0.13, *p* < 0.01). There was also a significant inverse relationship between total weekly efforts at max_>90%_ and the percentage change in eccentric hamstring strength, with a very small correlation shown (ρ = −0.08, *p* = 0.01). Total weekly efforts at max_>95%_ showed no relationship with the percentage change in eccentric hamstring strength (ρ = −0.02, *p* = 0.45).

**Figure 2:**
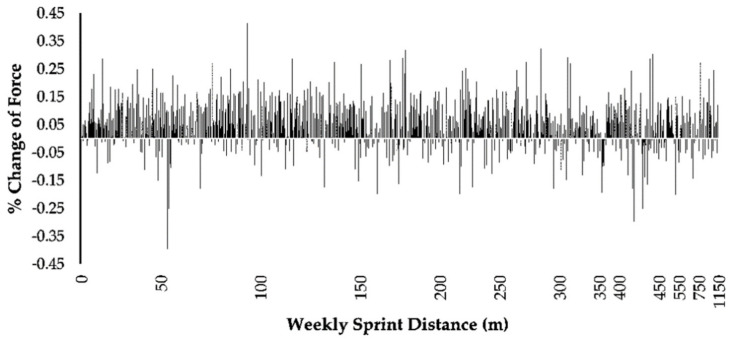
The mean percentage change in eccentric hamstring force in relation to total weekly sprint distance (m).

Mean percentage change in eccentric hamstring force was not significantly influenced by weekly sprint distance, F_(940,58)_ = 0.93, *p* = 0.66. As no apparent trend was shown between these factors, the optimal total weekly sprint distance could not be determined (Figure 2).

**Figure 3:**
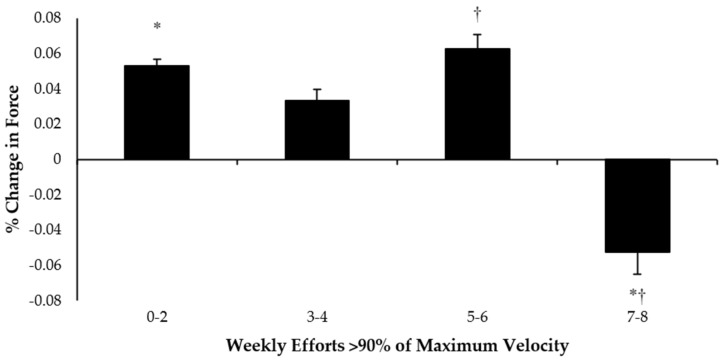
The mean percentage change in eccentric hamstring force in relation to weekly sprint efforts > 90% of maximum velocity (* denotes significance between 0–2 and 7–8 efforts; † denotes significance between 5–6 and 7–8 efforts, *p* < 0.05).

Mean percentage change in eccentric hamstring force was significantly influenced by weekly efforts > 90% of maximum velocity, F_(3,58)_ = 3.71, *p* = 0.01. Post hoc analysis showed that these differences occurred when comparing 7–8 sprint efforts with 0–2 sprint efforts (Δ = 0.11%; *p* = 0.03) and 5–6 sprint efforts (Δ = 0.12%; *p* = 0.03) (Figure 3).

## 4. Discussion

### 4.1. Main Findings

The aims of this study were to investigate the link between sprint load and eccentric hamstring strength, to establish optimal values of sprint load required to illicit a high eccentric force output, and to determine which of the factors studied had the largest influence on hamstring strength. We found that eccentric hamstring strength significantly decreased when 7–8 weekly sprint efforts at max_>90%_ were completed but not at <6 weekly efforts. Total weekly sprint distance or the weekly number of efforts completed at max_>95%_ were found to have no influence on eccentric hamstring strength. The number of maximal efforts and sprint distance required to illicit optimal levels of eccentric hamstring strength in professional football players could not be determined; however, we were able to establish the limit of weekly exposures at max_>90%_ before a decrease in hamstring force output occurred, which could place athletes at a greater injury risk [20,52].

### 4.2. Weekly Efforts at 90% of Maximum Velocity

In the professional football environment, we found an association between the number of weekly sprint exposures at max_>90%_ and eccentric hamstring strength. Interestingly, completing 7–8 efforts per week at max_>90%_ had a negative impact on eccentric hamstring strength; we could speculate that similar or greater decrements would be seen if >8 sprint efforts were performed in a week. These findings differ from previous studies investigating the effects of sub-maximal sprint efforts on injury risk, which have found that higher amounts of weekly maximal efforts are required before detrimental consequences occur [34,36]. These differences are likely because our study isolated and investigated one injury risk factor (eccentric hamstring strength), whereas, Malone et al. [34] and Colby et al. [36] studied “injury risk” as a whole, which can be multi-factorial and therefore difficult to quantify. With these previous research articles looking at multiple injury risk factors and encompassing all injuries, it may have been expected that fewer efforts would be required to have a negative impact on injury risk, however, that is not the case. Therefore, there must be another factor to consider to explain the different findings between these studies. One of the main differences between our study and the previous studies mentioned is that they involve different sports. In our study, football (soccer) players were monitored, as opposed to Gaelic football players [34] and Australian Rules footballers [36]. These three sports all differ with regards to their physical demands; in general, Gaelic football has the highest sprinting demands out of the three sports, where players complete approximately 44 sprint actions in a match [42]. The sprinting demands of Australian Rules football are similar to football (soccer), with approximately 29 and 17–36 in-match sprint actions completed, respectively [53,54,55]. Although the total number of sprint actions is similar, football players complete the majority of sprints over 0–10 m and only complete an average of 0.9–2.2 sprint efforts for distances greater than 20 m, whereas, higher sprint distances are found in Australian Rules football, likely due to the influence of the larger pitch dimensions found in the sport [56,57]. The low number of sprints completed in a football match at distances greater than 20 m may explain why no more than eight efforts per week at max_>90%_ were recorded in this study, as it typically takes >20 m to accelerate to this speed. Additionally, in this study the players were found to complete, on average, approximately one effort per week, therefore based on the ACWR it is of no surprise that eccentric hamstring force drops once a player has completed 7–8 efforts as these values are not being achieved on a regular basis to build up a tolerance to the chronic workload [58]. This observation is important for practitioners working in football to monitor the players’ weekly sprint efforts at max_>90%_, particularly in conjunction with each athlete’s ACWR.

### 4.3. Weekly Efforts at 95% of Maximum Velocity

It was observed in this study that football players completed only 14 efforts at max_>95%_ over the course of one-and-a-half playing seasons, corresponding to an average of 0.02 efforts per week. As the number of efforts at max_>95%_ were so low, the findings regarding efforts > 95% are tentative and may explain why no relationship was found between efforts at max_>95%_ and eccentric hamstring strength. This highlights why monitoring efforts at max_>95%_ may not be applicable in the practical setting of football, where using max_>90%_ would be more appropriate based on the physical demands of training and matches. Studies monitoring sprint efforts based on a percentage of a player’s maximal velocity in football are very limited; however, in Gaelic football it is found that players complete an average of seven ± four efforts at max_>95%_ in a week, with an average of four efforts completed during training and three efforts during matches [34].

The low number of efforts at max_>95%_ found in this study would suggest that it is very rare for football players to achieve such velocities; however, these findings may also be influenced by how data is recorded by the GPS devices. It is possible that players reach the required velocity to obtain an effort at max_>95%_, but these efforts may not be recorded by the GPS because of the dwell time set on the device. With the default dwell time being set at 0.6 s to account for errors in the sampling frequency [45], any occasions where the player reached an effort at max_>95%_ for less than 0.6 s would not be recorded. Additionally, although GPS devices with a 10 Hz sampling rate have been shown to measure velocity with high reliability, the accuracy of GPS devices can decrease at higher velocities when coupled with changes of direction, which would apply to team sports such as football, which involve many changes of direction during matches and training [59]. Nonetheless, the importance for sports practitioners to understand the accuracy of their sampling device(s) cannot be understated; where possible GPS with a sampling frequency ≥10 Hz is recommended, and while individual context may advocate an alternative dwell time than our suggested 0.6 s, selection of a shorter dwell time should be justified in any future reporting.

Straight-line sprinting plays a crucial role in match play, highlighting why it is important to supplement training with linear sprinting drills [9]. Therefore, it may be beneficial for practitioners to develop linear sprinting drills that also incorporate the technical and/or tactical aspects of the sport, which would increase training efficiency and also help to increase player motivation and effort [60]. However, with the accuracy of the data being questioned at velocities corresponding to max_>95%,_ the findings of this study further highlight that monitoring efforts at max_>90%_ is more appropriate in football and similar sports involving high-velocity running and changes of direction.

### 4.4. Weekly Sprint Distance

We found that weekly sprint distances did not influence eccentric hamstring strength. This finding was surprising considering the consistency of the data collected; to the best of our knowledge, no other study has investigated the relationship between weekly sprint distance and eccentric hamstring strength; however, a previous study reported the effects of weekly sprint distance, in football, in relation to injury risk as a whole [44]. In that study, trends were found between the weekly sprint distance completed and injury risk; however, they did not specify the associated injury sites. With the present study solely focussing on eccentric hamstring strength, it may be that any effect of weekly sprint distance on hamstring-specific strength is negligible. Alternatively, there could be a similar relationship between the weekly sprint distance and eccentric hamstring strength as there is with “injury risk” but the different findings in our study may be due to other factors. Malone et al. [44] reported trends between the weekly sprint distance and injury risk that were only apparent when considered independently of aerobic fitness and previous training load, but these factors play an important role in the risk of injury. This may be another reason that no association was found between the weekly sprint distance and eccentric hamstring strength in this study, as these factors were not considered independently of training load and aerobic fitness. Training load, in particular, seems to have a large impact on “injury risk”; it has been shown in previous studies that it was not necessarily weekly sprint distance that increased injury risk but actually rapid increases in acute workload in relation to the chronic workload [44,61]. These findings are highlighted in many other studies, which suggest that injury risk is also greatly affected by other external load measures, such as total distance, low intensity distance (<4 m/s), and the number of accelerations and decelerations [50,62,63,64,65]. Therefore, it is likely that any relationship between weekly sprint distance and “injury risk” is due to ‘spikes’ in external load based on the ACWR rather than decreases in eccentric hamstring strength.

### 4.5. Sprinting and Hamstring Strength Trends in Football

Many factors can affect physical output during training and matches. The manager/coaching staff’s preferred style of play can influence physical output—counter-attacking teams may show higher sprint performances compared with a possession-based team due to the quick transitional play associated with the former’s tactical roles. Additionally, the standard of quality between a team and their opposition affects high-intensity and sprint distance, with successful teams having to cover less sprint distance as the quality of opposition decreases [66]. A large gulf in quality can also have an effect on the inferior team’s movements, where if they are forced to play the majority of the match in their own half of the pitch, they may not have many opportunities to sprint [67]. Contextual factors and the influence of playing position on match running performance has previously been discussed in detail [68].

The players in this study were shown to have an average maximum velocity of 9.27 ± 0.27 m/s and an average weekly sprint distance of 212.1 ± 188.6 m. The maximum velocities of the players in this study were found to be lower than those in previous studies involving players in the English Premier League (9.55 m/s) and German national level (9.36 m/s) [55,69]. The lower velocities found in this study are possibly due to the higher level of standard associated with English Premier League and International players, with elite Norwegian players shown to have similar maximum velocities (9.2 m/s) as the players in this study, arguably because the standard of football is similar in the Scottish and Norwegian Leagues [70]. Although research is limited in providing weekly sprint distances that incorporate training and match data, the weekly sprint distance found in this study (212.1 ± 188.6 m) was lower than those previously reported for English Premier League players (298 m) [41]. As there are many factors that can influence the physical output produced by a team, it is important for practitioners to contextualise the results from this study and adjust their prescriptions based on the traits of the players/team being observed.

In accordance with previous studies looking into typical sprint distances during match-play, attackers were the quickest positional group and covered the greatest sprint distance per week, with defenders covering the least [67,71,72]. Although these studies did not include sprint distances during training, the majority of weekly sprint distances were obtained during a match and therefore had a large contribution to the weekly total [41]. Fullbacks/wide defenders typically cover high sprint distances, making it unusual that the ‘defenders’ group in our study, combining central and wide defenders, would still have the lowest average sprint distance between all positional groups. This finding suggests that either the central defenders recorded very low values so even the contribution of the fullbacks could not place them ahead of midfielders, who typically cover lower sprint distances than wide defenders, or this could also be due to the playing style of the football team, where fullbacks may not have been able to sprint as much based on the circumstances of the game and/or tactical responsibilities [67]. The addition of the fullbacks into the ‘defenders’ positional group did, however, seem to have an effect on the maximum velocities across the three positions, with defenders producing a higher average maximum velocity than the midfielders. With attackers shown to have the highest sprinting demands of the three positional groups, it may be expected that they were the most likely to obtain a hamstring injury, but this was not the case, with no clear trend found within outfield players based on their position [73]. Attackers, however, have been found to be more susceptible to a recurrent hamstring injury, with previous injury understood to be one of the largest contributing factors of re-injury [74,75,76]. The risk of re-injury in attackers is likely due to the structural integrity and strength of the hamstring muscles being compromised due to previous injury and therefore being affected by the high sprint demands of the position [77]; this suggests that hamstring strength plays a key role in reducing the risk of injury in football players, particularly for attackers.

It was found in this study that the players had an overall average eccentric hamstring force output of 427.47 ± 57.98 N and an average imbalance of 8.2 ± 6.65%. These findings exceed the recommended level, which suggest that strength greater than 337 N and a between limb imbalance less than 15% can reduce the risk of hamstring injury risk [52,78]. Hamstring strength, more so than limb imbalance, has been shown to have a greater influence on injury risk [20]. The manufacturers of the NordBord, Vald Performance, have presented the distribution of absolute peak bilateral force output results for over 21,000 NHE tests using the NordBord, involving teams from the English Premier League, English Championship and UEFA Champions League; it was found that the players involved in the current study have similar hamstring strength to those playing in the English Premier League (425 N) and slightly better strength scores than English Championship (418 N) and UEFA Champions League players (400 N), respectively [79]. These values would suggest that hamstring strength is not necessarily influenced by playing level, with arguably the highest stage of Club football (UEFA Champions League) displaying the lowest scores out of the three. Additionally, the physical demands are found to be very similar between the UEFA Champions League and English Premier League competitions. In an analysis completed by SkillCorner, the UEFA Champions League was shown to have similarities in the amount of average number of high-intensity and sprint activities and average sprint distance compared to the English Premier League. Differences were only found in the average peak sprint velocity, with the English Premier League displaying higher values [80]. In our study, a similar trend was found when hamstring strength was observed based on playing position. It was found that attackers had the highest strength scores, followed by defenders and midfielders, respectively. This trend corresponds with the respective maximum velocities of these positions, with strikers being the quickest, followed by defenders and then midfielders. These findings suggest that faster players tend to have stronger eccentric hamstring strength and would further highlight why, unexpectedly, very few trends are found showing attacking players to be more susceptible to hamstring injury, unless previously injured, as discussed above [73]. The greater hamstring scores associated with higher maximum velocities also correspond with the analyses conducted by SkillCorner and Vald Performance showing the English Premier League to have the highest average peak velocity and hamstring strength, respectively, even compared with the highest stage of Club competition—the UEFA Champions League. These trends suggest, therefore, that it is important for practitioners to ensure that the players with higher maximum velocities and sprint demands also correspond with having the highest eccentric hamstring strength values in relation to the squad average. Additionally, for those individuals it may be required to increase their minimum threshold well above the recommended level of 337 N to reduce hamstring injury risk.

### 4.6. Limitations and Practical Implications

As the study was observational, one of the limitations was that there were many occasions that data could not be collected for various reasons, for example: in weeks consisting of multiple matches, completing the NHE was not possible as the focus during that week would be on recovery and ‘muscle freshness’, and during international breaks, where players would either be with their respective nations or given time off. These are, however, common issues amongst most sports teams and therefore would be difficult to overcome [81]. It would also be worthwhile in future for practitioners to consider contextual factors, including match location, the quality of opposition, and match outcome, since these can impact running performance during matches [68], meaning that the number of sprint efforts during training could be responsively adjusted in order to achieve the targeted 7–8 weekly efforts at max_>90%_.

The overall objective of this study was to determine whether sprinting has an influence on eccentric hamstring strength in an attempt to reduce injury risk. Although it was established that eccentric hamstring strength is one of the risk factors in hamstring injury, this study did not directly measure injury risk. As there can be many influencing factors, this makes it difficult to conclude whether managing sprint loads will in fact reduce injuries; however, we know that to reduce the risk of injuries we must mitigate the factors involved. As we were able to establish that performing 7–8 weekly efforts at max_>90%_, significantly reduces eccentric hamstring strength, one can imply that there is also an increased risk of hamstring injury when reaching this number of weekly efforts, based on hamstring strength being a risk factor of injury. However, to better understand the injury risk associated with sprinting, it would also be beneficial to study its effects on other risk factors for hamstring injury, allowing practitioners to identify all the mediators of injury and consider preventative strategies accordingly.

It has been established by previous research that practitioners should dose players with maximal effort sprints throughout the training week and the findings of this study suggest that they can be confident that obtaining >90% of each player’s maximal velocity is beneficial in conditioning the hamstring muscles and maintaining their strength. However, careful monitoring is required to ensure that players do not exceed 7–8 efforts per week to maintain their eccentric hamstring strength levels. There is a possibility that these values are influenced by the ACWR; therefore, it would be beneficial for future studies to investigate a link between the ACWR, weekly efforts at max_>90%_ and hamstring strength to identify whether different thresholds are found based on different chronic loads. However, limitations in the methods of calculation and the influence of various contextual factors on weekly load must be taken into consideration when applying the ACWR within the professional environment [82,83]. To adopt these findings in other sports, practitioners may need to tailor their prescribed sprint loads based on the physical demands of their sport and the chronic loads of their athletes, where sports with less sprint demands may have a lower threshold of efforts at max_>90%_, before eccentric hamstring strength decreases.

## 5. Conclusions

We have shown that eccentric hamstring strength significantly decreases when professional football players complete 7–8 weekly sprint efforts at max_>90%_, but total weekly sprint distance or the weekly number of sprint efforts completed at max_>95%_ have no influence on eccentric hamstring strength. The reason that no relationship was found between eccentric hamstring strength and sprints at max_>95%_ was largely due to the limited number of efforts recorded, making it difficult to conclude whether there was any additional benefit to exposing players to efforts greater than 95% of their maximum velocity. Based on the physical demands of football during training and matches and the uncertain GPS accuracy at maximal velocities, it is suggested that practitioners use max_>90%_ when monitoring training and match load, ensuring that players do not exceed 7–8 efforts per week to maintain good eccentric hamstring strength levels, thereby reducing the risk of potential injury.

## Figures and Tables

**Table 1:** Profiles of participants (Mean ± SD).

Playing Positions *	Age (Years)	Height (cm)	Mass (kg)	Body Fat (%)	Yo-Yo IE2 † Distance (m)
Defenders (*n* = 21)	21.7 ± 4.2	183.4 ± 5.7	78.9 ± 8.2	11.2 ± 2.2	2027 ± 714
Midfielders (*n* = 17)	21.5 ± 5.4	177.7 ± 5.3	71.1 ± 8.1	11.2 ± 2.3	2096 ± 808
Attackers (*n* = 20)	22.2 ± 4.6	181.3 ± 7.0	78.4 ± 10.7	11.3 ± 3.3	1875 ± 938
Squad (*n* = 58)	21.8 ± 4.6	181.0 ± 6.4	76.5 ± 9.6	11.2 ± 2.6	2002 ± 442

* Positions were determined by where the majority of playing time occurred throughout the study. † Yo-Yo IE2 = YoYo-Intermittent Endurance Level 2 Test.

**Table 2:** Sprinting and hamstring strength profiles of players using GPS and NordBord data, respectively. Data are presented based on the players’ playing positions and as a collective group (Mean ± SD).

Position	Maximum Velocity (m/s)	Weekly Sprint Distance (m)	Weekly Efforts > 90% of Max Velocity (*n*)	Weekly Efforts > 95% of Max Velocity (*n*)	Hamstring Strength (N)	Strength Imbalance (%)
Defenders	9.30 ± 0.24	204.8 ± 178.8	1.12 ± 1.51	0.02 ± 0.14	420.14 ± 52.84	7.90 ± 7.35
Midfielders	9.19 ± 0.23	208.4 ± 193.2	0.86 ± 1.24	0.01 ± 0.10	416.15 ± 48.80	9.87 ± 6.32
Attackers	9.32 ± 0.30	224.7 ± 195.3	0.87 ± 1.37	0.02 ± 0.18	446.98 ± 66.42	6.99 ± 5.75
Overall Squad	9.27 ± 0.27	212.1 ± 188.6	0.96 ± 1.39	0.02 ± 0.14	427.47 ± 57.98	8.20 ± 6.65

## Data Availability

The data presented in this study are available on request from the corresponding author.
